# Borderline Brenner tumor of the ovary: a case report with immunohistochemical and molecular study

**DOI:** 10.1186/s13048-014-0101-7

**Published:** 2014-10-29

**Authors:** Rossella De Cecio, Monica Cantile, Francesca Collina, Laura Marra, Clemente Santonastaso, Cono Scaffa, Gerardo Botti, Nunzia Simona Losito

**Affiliations:** Pathology Unit, Istituto Nazionale Tumori Fondazione “G. Pascale”, via Mariano Semmola, 80131 Napoli, Italy; Department of Gynecologic Oncology, Istituto Nazionale Tumori Fondazione “G. Pascale”, via Mariano Semmola, 80131 Napoli, Italy

**Keywords:** Brenner tumors, Immuno-profile, Molecular analyses

## Abstract

**Background:**

Borderline Brenner tumor of the ovary is a rare entity characterized by papillary structures with a fibro-vascular core, covered by a transitional epithelium, and by the absence of stromal infiltration. It is associated, by definition, with a benign component of Brenner tumor.

**Case:**

We report a case of a 68-year-old woman, with a right ovarian mass, whose morphology and immuno-profile were consistent with the diagnosis of a borderline Brenner tumor. Immunohistochemistry carried out on selected markers may help to formulate the diagnosis, more than the molecular analyses.

**Electronic supplementary material:**

The online version of this article (doi:10.1186/s13048-014-0101-7) contains supplementary material, which is available to authorized users.

## Background

Transitional cell tumors of the ovary, described for the first time by Brenner in 1907, are rare neoplasms and account for about 2% of all ovarian tumors.

Transitional cell tumors, in turn, include two distinct clinic-pathological subtypes: Transitional cell carcinomas (TCCs) and Brenner Tumors [1].

The World Health Organization (WHO) classifies Brenner tumors into three categories: benign, borderline and malignant [2]. Borderline Brenner tumors, usually associated with a benign Brenner tumor, are characterized by papillary structures with a fibro-vascular core covered by a transitional epithelium.

Borderline Brenner tumors are negative for p16, Rb and p53, and show weak immunostaining for Cyclin D1, moderate for Ras and strong for EGFR. They also express p63, as in benign subtypes but not in malignant ones, and present a diffuse staining for CK7, CA125, thrombomodulin and EMA, as in all subtypes of Brenner tumors [3]. Malignant Brenner tumor is also negative for p16, Rb and p53, but strongly positive for EGFR, Cyclin D1 and Ras. Over-expression of p53 and p16 and negative immunoreaction for EGFR, Cyclin D1 and Ras can be considered for differential diagnosis with TCCs [4].

More recently, molecular analyses have revealed a number of molecular alterations that allowed to understand the molecular pathways involved in the pathogenesis and progression of Brenner Tumors. In particular, the loss of p16 expression in borderline and malignant Brenner tumors seems to be associated with promoter hyper methylation and a homozygous deletion [5]. Moreover, several KRAS and PIK3CA somatic mutations have been identified in about 30% of borderline Brenner tumors [5].

We report the case of a 68-year-old woman with a right ovarian mass, for which the morphology and immunohistochemistry (IHC) analyses were consistent with the diagnosis of borderline Brenner tumor. Molecular analyses were also performed to evaluate the methylation status of p16 and mutational status of KRAS and PIK3CA. Furthermore, since any activating mutation associated with EGFR over-expression has been described in the literature, we also evaluated the mutational status of the gene and verified EGFR copies number gain or amplification.

## Case presentation

A 68-year-old woman was admitted to gynecological surgery of our Institute for the presence of a suspect right ovarian mass. After a careful medical history, we discovered that the patient had been operated 12 years before for a neoplastic lesion in the left colon, with a diagnosis of “moderately differentiated adenocarcinoma infiltrating the muscularis B1 sec. Dukes, pT2 N0 G2”. No history of endometriosis was reported.

**Figure 1:**
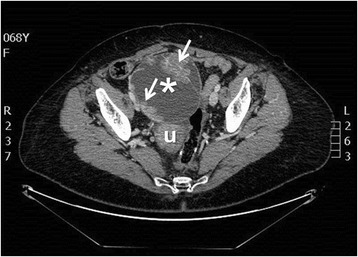
**Contrast-enhanced CT scan showing a complex and heterogeneous pelvic mass with inner vegetations and septations in the anatomic site of the right adnexa (max diameter 17 cm), not dissociable from uterus and contiguous bowel (asterisk: pelvic mass; arrows: vegetations and septations; u: uterus).**

The patient complained of abdominopelvic pain for two months. Vaginal examination revealed a solid mass in the right adnexal site attached to pelvis and uterus (diameter 15 cm Ø approximately) and CA125 levels were slightly increased (49.4 IU/ml). Contrast-enhanced CT scan of abdomen and pelvis revealed a “complex” pelvic mass (with both cystic and solid components) in the anatomical location of the right adnexa (maximum diameter 17 cm), not dissociable from uterus and contiguous bowel, with slight peritoneal effusion (Figure 1).

**Figure 2:**
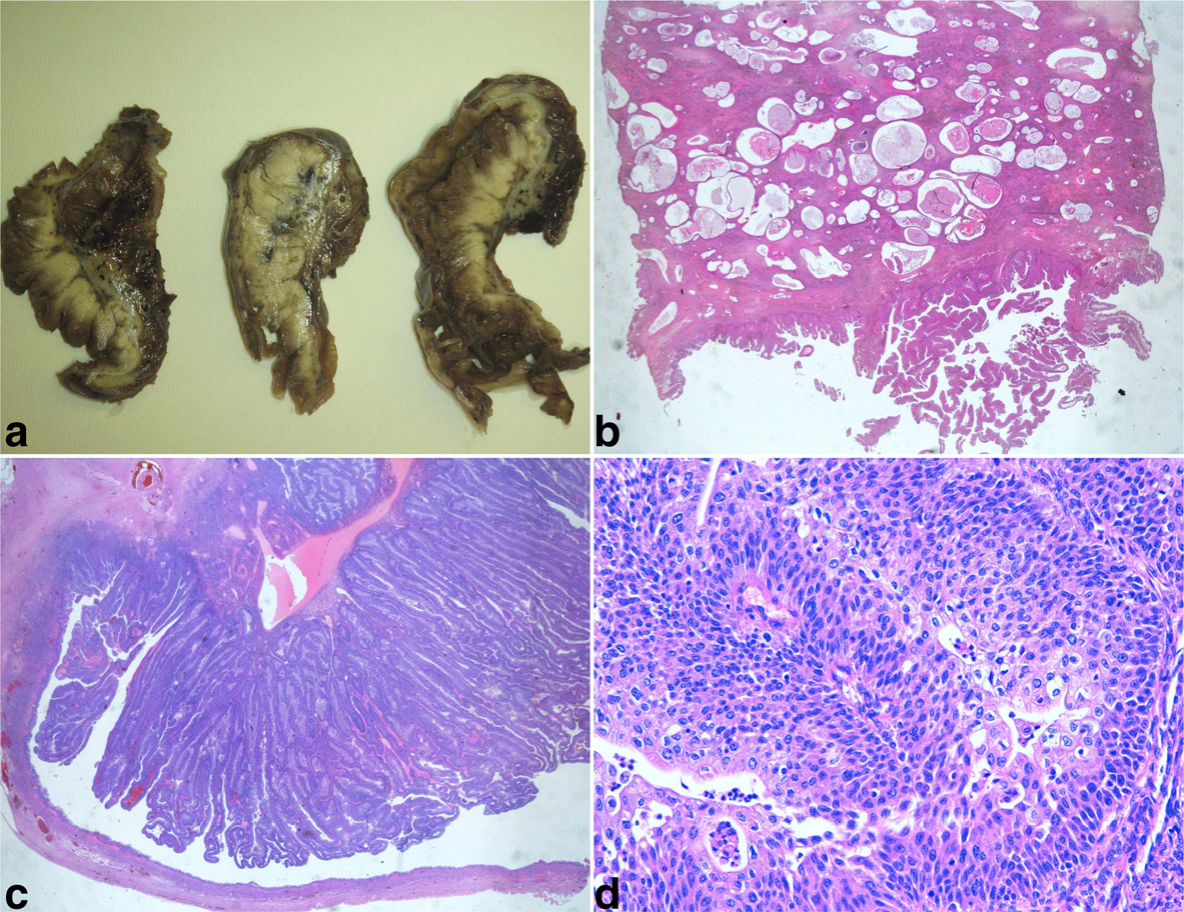
**Gross and microscopic description of Brenner tumor: a)** Ovarian mass with solid papillary component; **b)** H & E representative of a solid adeno-fibromatous component (5X); **c)** H & E representative of cystic formations lined by mucinous columnar epithelium and by papillary transitional cellular component (10X); **d)** H & E representative of transitional epithelial cells with pale cytoplasm, indented nuclei and mild nuclear atypia (40X).

At laparotomy, there was a right adnexal tumor (maximum diameter 15 cm) with deep uterine and visceral adhesions; a total hysterectomy with right salpingo-oophorectomy and infracolic omentectomy was performed (Figure 2).

Microscopically, the lesion was characterized by a solid adenofibromatous component with solid nests of transitional epithelial cells with pale cytoplasm, indented nuclei and cystic formations lined by mucinous columnar epithelium and by an exuberant papillary transitional cellular component with mild nuclear atypia (Figure 2).

The definitive pathological examination showed a Brenner borderline ovarian tumor with atypia (FIGO stage IA) and no adjuvant treatment was recommended. The patient status was NED (no evidence of disease) at 12-month follow-up visit.

**Figure 3:**
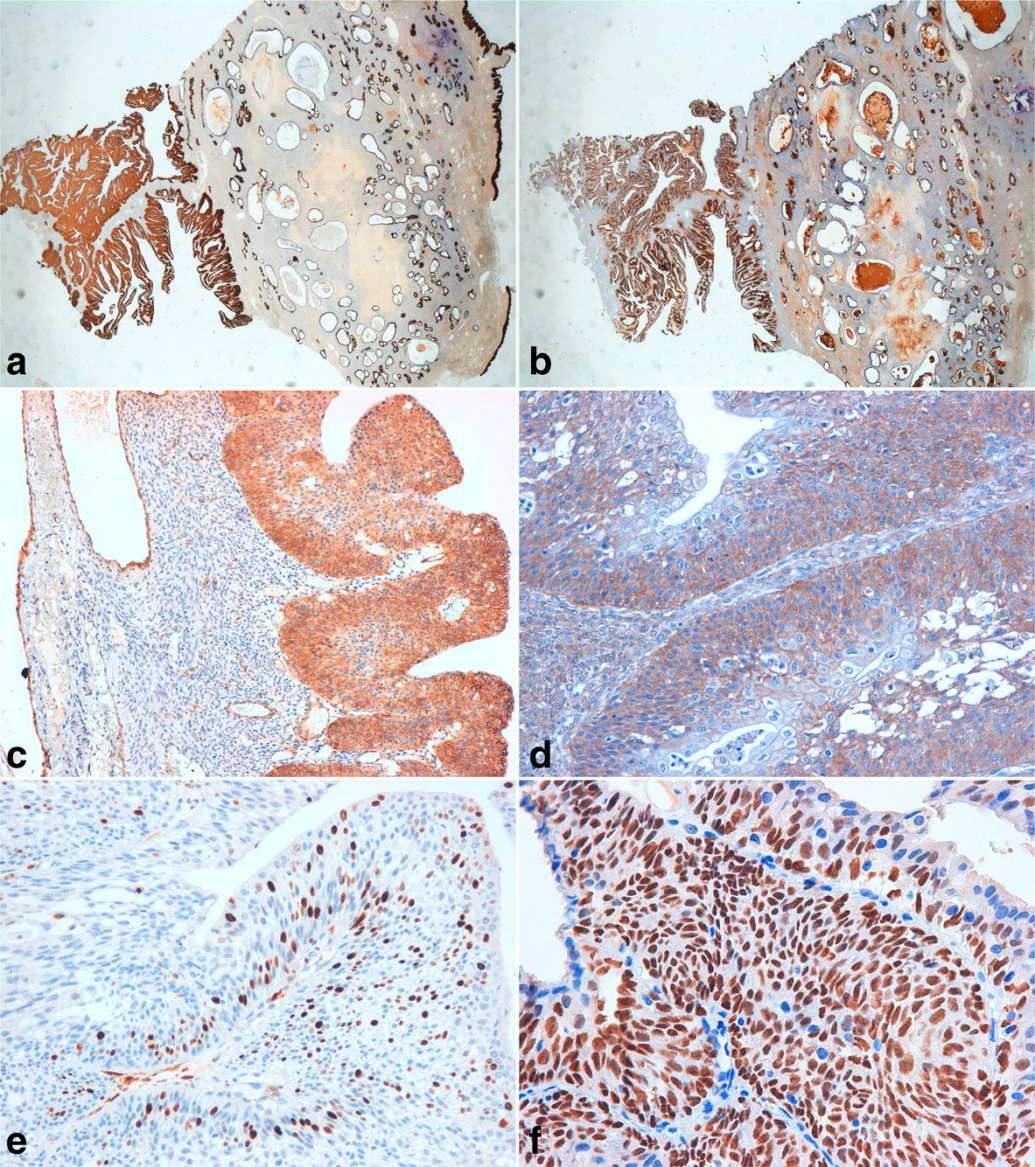
**Borderline Brenner tumor immunoprofile. a)** positive immunostaining for CK7 (10X); **b)** positive immunostaining for EMA (10X); **c)** positive immunostaining for thrombomodulin (20X); **d)** positive immunostaining for EGFR (20X); **e)** positive immunostaining for p16 (40X); **f)** positive immunostaining for p63 (20X).

IHC in transitional cells was strongly positive for CK7, CA125, thrombomodulin and EMA, and negative for CDX2, CK 20 and p53 (Additional file 1: Figure S1). Moreover, transitional papillary epithelial cells showed a strong positivity for p63 and EGFR, while p16 appeared positive in the basal, parabasal and intermediate layer of exophytic papillary epithelial component (Figure 3).

In order to find p16 promoter hyper-methylation, molecular analysis was carried out using a commercial kit (Methylation Kit, Diachem, Naples, Italy) and it showed the absence of molecular alteration. Mutational analyses of KRAS and PK3CA were performed by direct sequencing, after specific PCR amplification, as previously reported [3]. The analyses did not reveal the presence of the mutations described in literature (data not shown). Finally, mutational analysis of EGFR was performed by a commercial kit (EGFR Mutation Analysis Kit, EntroGen, Tarzana, CA, USA), containing a distinct primer/probe mix in order to detect, by Real Time PCR system, all EGFR point mutations described in literature (Exons 18, 19, 20, 21). No activating mutations were present in our sample.

**Figure 4:**
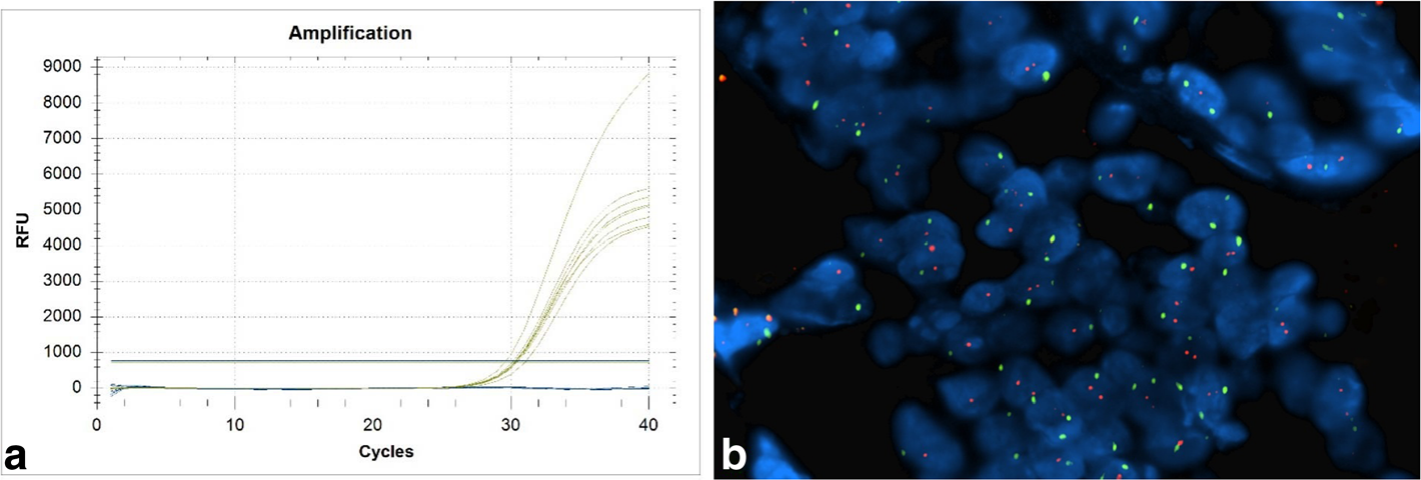
**EGFR molecular analyses. a)** Absence of mutations detected by RT-PCR method (the yellow increased curves indicate the internal controls of the kit, the blue flat lines indicate our sample); **b)** Absence of chromosomal aberrations detected by FISH method (red signals represent the EGFR gene and green signals represent the centromeric sequence in chromosome 7. The above picture shows a balanced disomy (each nucleus with 2 red and green signals, ratio = 1; Magnification, ×60)).

Beside, FISH analysis (LSI EGFR SpectrumOrange/CEP 7 SpectrumGreen Probe, Vysis Abbott) did not reveal gene amplification or gene copies number gain (Figure 4).

However, morphology and immuno-profile of the sample were consistent with the diagnosis of a borderline Brenner tumor of the ovary.

## Discussion

Brenner tumors of the ovary are rare cancers. Up to date, about 30 cases of Brenner borderline tumors have been described, all characterized by the absence of stromal infiltration and, by definition, associated with a benign component [6]. These lesions seem to originate from transitional metaplasia of the tubal epithelial cells [7]. As for low-grade ovarian serous carcinomas, the acquisition of a series of molecular alterations lead to its evolution from a benign lesion [8].

Our case of borderline Brenner tumor is morphologically characterized by papillary projections and solid nests of transitional cells surrounded by dense fibrous tissue.

Transitional cells showed only mild, focal nuclear atypia and low mitotic index; extensive sampling ruled out infiltration. Therefore, our diagnosis was “Brenner Borderline tumor with atypia” [9].

We performed an immunohistochemical study to demonstrate the origin of the tumor from the ovary and then to exclude that it was a metastasization from the bowel.

The immunohistochemical panel showed strong positivity for p63 and EGFR in the epithelial component of the tumor, while p16 was positive in the basal, parabasal and intermediate layer of the papillary epithelial component.

We also found, in transitional cells, a strong cell positivity for CK7, CA125, thrombomodulin and EMA, confirming the ovarian origin, and a negativity for CDX2 and CK 20. These latter findings excluded, immunophenotypically, the presence of metastasis from the primary tumor of the colon, dating back 12 years earlier.

In the light of recent findings on the role of some molecular markers in the pathogenesis and progression of this tumor [5], we have also carried out molecular analyses to verify the methylation of p16 promoter and mutational status of KRAS, PIK3CA and EGFR. For the latter we have also looked for potential aberrations at gene/chromosome level. None of the performed molecular analyses showed positive results and, above all, they did not provide any important support to the histomorphological diagnosis.

## Conclusions

Our case can be placed among the low-grade lesions, based on morphology and immunophenotype. As described for papillary serous ovarian tumors of low-grade, the presence of specific markers has been of fundamental support for the differential diagnosis with benign (low expression of EGFR and p16) and malignant (negative for p16) Brenner tumors, and with TCCs (negative for EGFR).

However, the molecular alterations described in literature, currently do not seem to be so specific as to suggest the possibility of developing targeted biological therapies for these lesions. Therefore, it would be necessary to better characterize these lesions, from a molecular point of view. Although they do not show a tendency to evolve into more aggressive malignancies, very little is known about cell biology that determines its pathogenesis.

## Consent

Written informed consent was obtained from the patient for publication of this Case Report and accompanying images. A copy of the written consent is available for review by the Editor-in-Chief of this journal.

## Additional file

Additional file 1: Figure S1. Negative immunostaining for p53 in Borderline Brenner tumor (20X).
