# Supplementary figures and images for: Borderline Brenner tumor of the ovary: a case report with immunohistochemical and molecular study
Source: J Ovarian Res. 2014 Oct 29;7:101. doi: 10.1186/s13048-014-0101-7 (PMC4226905; doi:10.1186/s13048-014-0101-7)

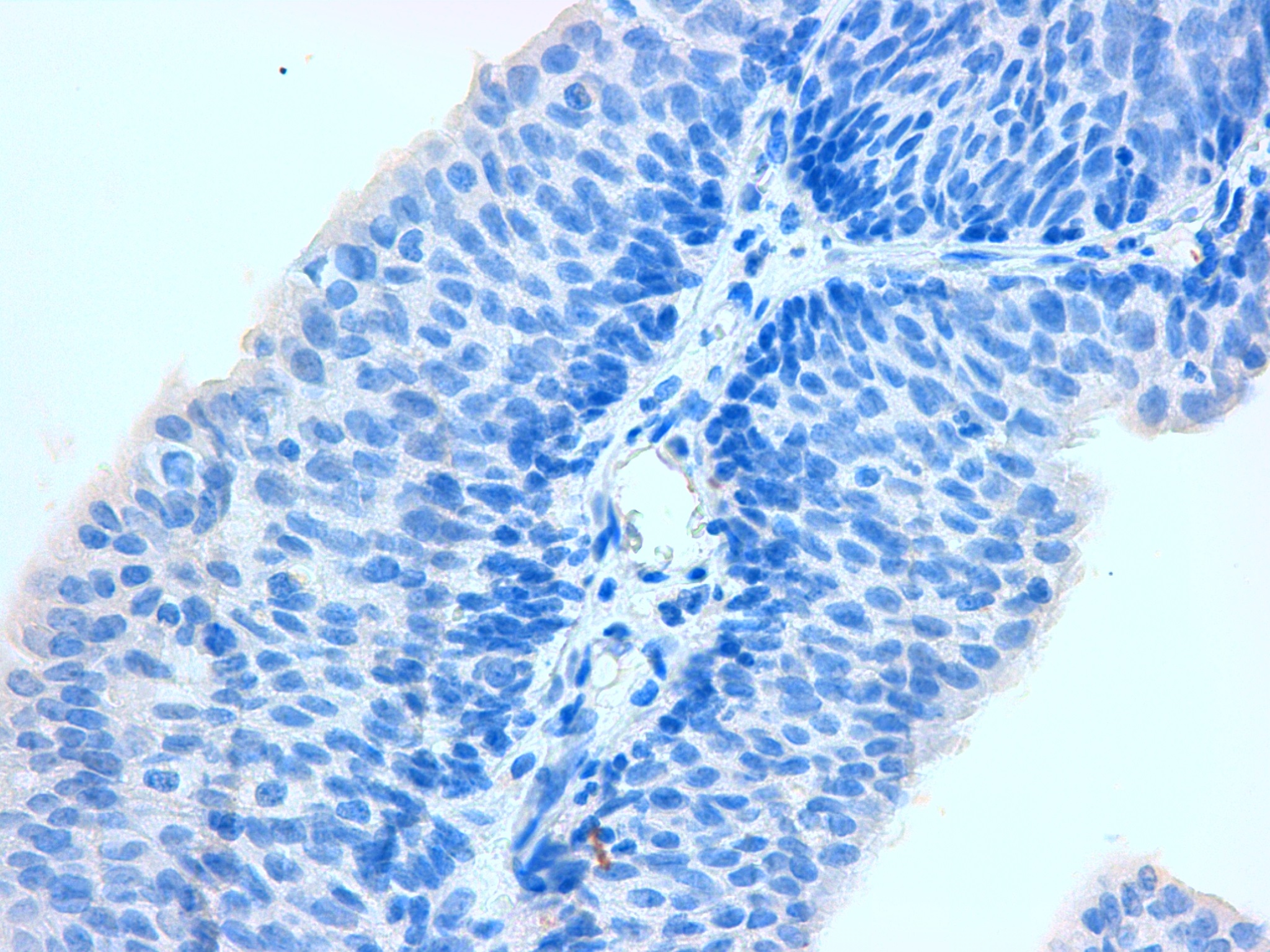

Supplement: Additional file 1: Figure S1. — Negative immunostaining for p53 in Borderline Brenner tumor (20X). [file 13048_2014_101_MOESM1_ESM.jpeg]
